# An ICT-Based Coumarin Fluorescent Probe for the Detection of Hydrazine and Its Application in Environmental Water Samples and Organisms

**DOI:** 10.3389/fbioe.2022.937489

**Published:** 2022-06-14

**Authors:** Xina Liu, Meiqing Zhu, Chenyang Xu, Fugang Fan, Panpan Chen, Yi Wang, Dongyang Li

**Affiliations:** ^1^ Anhui Provincial Key Laboratory of Quality and Safety of Agricultural Products, College of Resources and Environment, Anhui Agricultural University, Hefei, China; ^2^ School of Chemical and Environmental Engineering, Anhui Polytechnic University, Wuhu, China; ^3^ Laboratory of Agricultural Information Intelligent Sensing, College of Biosystems Engineering and Food Science, Zhejiang University, Hangzhou, China

**Keywords:** hydrazine, detection, density functional theory, imaging, fluorescent probe

## Abstract

As an inorganic small molecule pollutant, the toxicity and potential carcinogenicity of hydrazine (N_2_H_4_) are of increasing concern. In this work, A water-soluble fluorescent probe (OCYB) based on the intramolecular charge transfer (ICT) mechanism for the detection of hydrazine was designed and synthesized. Taking the advantage of 4-bromobutyryl as the recognition group, the high selectivity of OCYB to N_2_H_4_ was confirmed by steady-state fluorescence spectroscopy. The limit of detection (LOD) was calculated to be 78 nM in the DMSO-HEPES (pH 7.4) system. The detection mechanism was verified by NMR, HRMS and density functional theory (DFT) calculations. In addition, OCYB exhibits strong anti-interference ability and an “Off-On” fluorescence enhancement effect. Importantly, OCYB can be used to effectively monitor the fluorescence distribution of N_2_H_4_ in environmental water samples and organisms.

## Introduction

Hydrazine (N_2_H_4_) is a corrosive and highly reductive fine chemical raw material that is widely used in pharmaceuticals, chemistry, catalysis, agriculture, and other fields (Zhang et al., 2018; Wang et al., 2021). It is also used as a raw material for drugs, pesticides, dyes, foaming agents, contrast agents, antioxidants, and foaming agents (Zhang et al., 2020b). Despite its wide industrial applications, hydrazine is highly toxic and carcinogenic (Chen et al., 2021; Cui et al., 2022). Although not endogenous, trace spills during use or transportation can pollute the environment and enter the body by inhalation or skin contact, causing respiratory and neurological harm, including eye and skin irritation, liver, kidney, and central nervous system damage (Zhang et al., 2020a). At present, N_2_H_4_ has been classified as a carcinogen by the U.S. Environmental Protection Agency (EPA) with a threshold limit value (TLV) of 10 ppb (Xu W.-Z. et al., 2018; Yan et al., 2021). As the use of hydrazine increases, the risks become increasingly serious. The hazards of hydrazine are gradually being recognized and noticed. The development of a speedy, simple, sensitive, and selective method for detecting hydrazine is critical in the realm of environmental and biological sciences.

Many methods for the routine detection of hydrazine have been reported in recent years, such as titrimetric, spectrochemical, electrochemical, and surface-enhanced Raman spectroscopy, and chromatographic methods (Xu H. et al., 2018; Zhu et al., 2021b; Hu et al., 2021). Bo et al. developed a Pd/MCV composite electrode material for the electrochemical detection of hydrazine hydrate with a detection limit of 1.49 × 10^–10^ mol/L. Gu et al. used surface-enhanced Raman spectroscopy (SERS) to detect hydrazine in water with a detection limit of 8.5 × 10^–11^ mol/L (Gu and Camden, 2015). However, these assays have several drawbacks, such as the requirement for pretreatment of test samples, the preparation of reagents for testing, extended testing times, and sophisticated testing instruments (Guo et al., 2019; Qu et al., 2019). Furthermore, several of these assays have low sensitivity, are unable to detect low hydrazine concentrations, and cannot be utilized to detect hydrazine in biological samples (Chen et al., 2020; Jiang et al., 2020; Zhu et al., 2022). The advantages of fluorescence analysis include small sample size, great sensitivity, good reproducibility, speed, and convenience, among others (Yang et al., 2017; Zhu et al., 2019; Han et al., 2020; Yin et al., 2021). Fluorescence probe technology has advanced rapidly in both theory and application, and it is now widely employed in a variety of sectors including medicine, life science, and environmental science (Li et al., 2017; Yan et al., 2020; Cao et al., 2021). Many of these probes, however, still have issues including low selectivity, biocompatibility, and background interference, which severely limit their use in cells and *in vivo* (Na et al., 2016; Wang et al., 2017; Ye et al., 2021). Great structural modifiability, changeable excitation and emission wavelengths, high sensitivity, low cytotoxicity, and facile metabolic breakdown are all advantages of organic small-molecule fluorescent probes (Zhu et al., 2020; Zhu et al., 2021a; Ksenofontov et al., 2022). Therefore, it is significant to design and synthesize organic small-molecule fluorescent probes that are selective, sensitive, biocompatible, and capable of detecting hydrazine in cells and *in vivo*.

Coumarins are fluorophores with the parent ring structure of benzopyrone, and they are widely used as fluorophores for laser dyes, fluorescent brighteners, small-molecule fluorescent probes, and other applications because of their high fluorescence quantum yield, good light resistance, large Stokes shift, and adjustable optical properties. Coumarin does not glow by itself, but it can be changed with a variety of sites, such as inserting different types of groups at different places or linking multiple aromatic rings to increase the conjugation system, resulting in fluorescent dyes with a variety of emission bands. In the present study, a new recognition group was designed by the introduction of 7-hydroxycoumarin due to its excellent spectral performance and stability. 4-Bromobutyryl protected hydroxycoumarin to quench its fluorescence and improve the sensitivity and selectivity of the probe (Zhang et al., 2021). A 2-oxo-2H-chromium-7-yl 4-bromobutyrate (OCYB) is a relatively new probe designed and synthesized based on the intramolecular charge transfer (ICT) mechanism, which can detect N_2_H_4_ and exhibits specific recognition ability, strong anti-interference ability, and low detection limit. In addition, OCYB can also detect N_2_H_4_ in water samples and has been successfully applied to cell and zebrafish imaging.

## Experimental Section

### Reagents and Instruments

The experiments were carried out using an Agilent Cary Eclipse fluorescence spectrophotometer (Santa Clara, CA, United States ) equipped with a xenon lamp and a quartz cuvette with a volume of 3.0 ml. The UV-Vis absorption spectra were performed on a Shimadzu UV-1900i spectrophotometer (Kyoto, Japan). pH values were obtained with a Shanghai Rex pH -25 digital display acidity meter (Shanghai, China). All nuclear magnetic resonance (NMR) spectra were recorded using an Agilent 600 MHz DD2 (DirectDrive2) spectrometer (Santa Clara, CA, United States ). Analytical data for high-resolution mass spectrometry (HRMS) was collected with an Agilent Precision Mass Time of Flight Mass Spectrometer 6,520 mass spectrometer equipped with an electrospray ionization source. Hela cells and zebrafish imaging were investigated with an EVOS fluorescence auto-inverted fluorescence microscope (Waltham, MA, United States ) and a Nikon inverted fluorescence microscope (Tokyo, Japan), respectively.

N_2_H_4_, 4-bromobutyryl chloride and triethylamine were purchased from Bailingwei Technology Co. Ltd (Beijing, China), Ciensi Biochemical Technology Co., Ltd. (Tianjin, China) and Aladdin Reagent Co., Ltd (Shanghai, China). Tryptophan (Try), tyrosine (Tyr), arginine (Arg), histidine (His), lysine (Lys), aspartic acid (Asp), threonine (Thr), glutamic acid (Glu) and glycine (Gly) were obtained from Sinopharm Chemical Reagent Co., Ltd (Shanghai, China). Other reagents were all obtained from Xilong Chemical Co., Ltd. (Guangdong, China). All chemical reagents are directly used in the experiment without further purification. Deionized water (18.25 MΩ cm) from the Milli-Q® Direct 8 & 16 Ultrapure Water System (Billerica, MA, United States) was used to prepare the aqueous solution for the experiment. All glass containers are rinsed three times with deionized water before use.

### Synthesis and Characterization of OCYB

**SCHEME 1:**
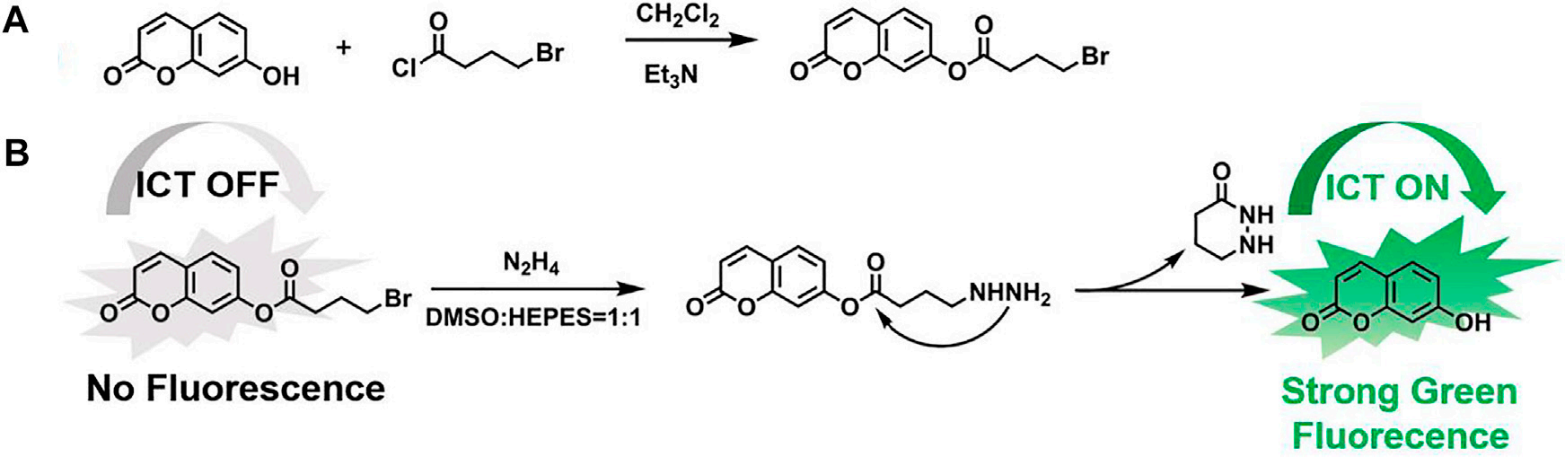
**(A)** Synthetic route of OCYB and **(B)** Proposed mechanism for discriminative detection of N_2_H_4_.

The coumarin-like fluorescent probe OCYB was produced using a theoretically derived approach (Scheme 1A). 7-Hydroxycoumarin (2.0 g, 12.3 mmol) and triethylamine (1.87 g, 18.5 mmol) were mixed in CH_2_Cl_2_ and cooled to 0 °C. Then, 4-bromobutyryl chloride (2.73 g, 14.8 mmol) was slowly added to the solution using a constant pressure dropper and the mixture was stirred at room temperature for 3 h. After the concentration under reduced pressure, the crude product was purified by chromatography into 2-oxo-2H-chromium-7-yl 4-bromobutyrate and the probe OCYB was obtained (3.2 g, 83.6%). ^13^C NMR (151 MHz, DMSO-*d*_*6*_) δ 170.96, 160.08, 154.53, 153.24, 144.23, 129.72, 119.01, 115.99, 110.46, 34.21, 32.65, 28.09, 27.91. ^1^HNMR (600 MHz, DMSO-*d*_*6*_) δ 8.05 (d, J = 9.6 Hz, 1H), 7.75 (d, J = 8.4 Hz, 1H), 7.28 (d, J = 2.2 Hz, 1H), 7.16 (dd, J = 8.4, 2.2 Hz, 1H), 6.45 (d, J = 9.6 Hz, 1H), 3.62 (t, J = 6.6 Hz, 2H), 2.76 (t, J = 7.3 Hz, 2H), 2.19 – 2.15 (m, 2H).

### Optical Properties of OCYB

Spectroscopic analysis was performed in a mixture of DMSO and 2-[4-(2-hydroxyethyl)-1-piperazinyl] ethanesulfonic acid HEPES (1:1, v/v, pH = 7.4). The probe OCYB was dissolved in DMSO and made into a 10 mM stock solution; the analytes AgNO_3_, Ca(NO_3_)_2_, NaNO_3_, KNO_3_, NaCl, CoCl_2_, BaCl_2_, NiCl_2_, HgCl_2_, and amino acids Try, Cys, Tyr, His, Glu, Asp, Thr, Ary were configured into a 5 × 10^–3^ M solution. After the addition of the analytes, the spectra of UV-vis and fluorescence were recorded using a 3 ml volume quartz cuvette. The conditions were set as follows: *E*_x_ = 330 nm, *E*_m_ = 350–700 nm.

### Cell Culture and Cellular Imaging

HeLa cells were cultured in 96-well plates for 24 h at 37°C. A series of probe solutions (10, 20, 30 and 40 μM) in a concentration gradient was added to 96-well culture plates containing HeLa cells (5 × 10^–4^ cells per well incubated), and untreated HeLa cells were used as controls. After 24 h of incubation, the cell viability of HeLa cells was determined by the CCK-8 assay.

Untreated HeLa cells were used as control. The wells of the plate containing Hela cells were incubated for 24 h and then treated for 30 min with N_2_H_4_ solution (50 μM). Then, HeLa cells were treated with OCYB (20 μM) for 20 min in a constant temperature incubator and washed three times with PBS solution to remove the excess N_2_H_4_. Finally, untreated HeLa cells and treated HeLa cells were imaged using an Eclipse TiS fluorescence microscope (Nikon, Japan).

### Imaging of Zebrafish

Zebrafish eggs were incubated in an E3 medium containing nutrient salts (sodium bicarbonate, CaCl_2_•2H_2_O, MgSO_4_•7H_2_O and KCl) and placed at a constant temperature incubator at 28°C. Normally growing 3d zebrafish were selected for the experiment. The zebrafish were first incubated in the E3 medium with N_2_H_4_ solution (final concentration of 100 μM) for 30 min and then treated with OCYB (20 μM) for 30 min. Zebrafish without N_2_H_4_ was used as blank control. Zebrafish were anesthetized with tricaine (50 mg/L) during the experiment and imaged using an Olympus BX41-32P02-FLB3 fluorescence microscope (Tokyo, Japan).

### Treatment of Water Samples

Water samples for this experiment were collected from rivers around parks and schools in the Hefei area of Anhui, China, as test water samples. The same concentration of HEPES buffer was prepared separately with different water samples. Then N_2_H_4_ was added at 0.1, 0.5, and 1 mg/L. The OCYB probe solution was incubated with the samples for 1 h, and the fluorescence spectra of each sample were recorded (*E*_x_ = 330 nm). The intercept of the standard addition curve was used to calculate the concentration of N_2_H_4_ in the raw water samples.

## Results and Discussion

### Structural Characterization of OCYB

Because of its excellent spectroscopic properties and stable absorption, OCYB was designed and synthesized using the intramolecular charge transfer (ICT) mechanism by introducing the 4-bromobutyryl group as a recognition group in 7-hydroxycoumarin, and the synthetic route for high-yield OCYB is shown in Scheme 1A. The possible recognition mechanism of probe OCYB to N_2_H_4_ is shown in Scheme 1B. The ICT process is disturbed and the probe OCYB is not fluorescent because the hydroxyl group in the probe OCYB is shielded by 4-bromobutyryl. When N_2_H_4_ is introduced to the probe OCYB, it works as a nucleophilic reagent, attacking the 4-bromobutyryl group and undergoing a substitution-cyclization reaction, resulting in a “turn-on” phenomenon and green fluorescence. The apparent change in fluorescence intensity before and after the interaction of the probe OCYB with N_2_H_4_ was used to specifically identify and detect N_2_H_4_. This was confirmed by ^1^H NMR, ^13^C NMR and HRMS (Supplementary Figure S1-3).

### Optimization of Reaction Conditions

**FIGURE 1:**
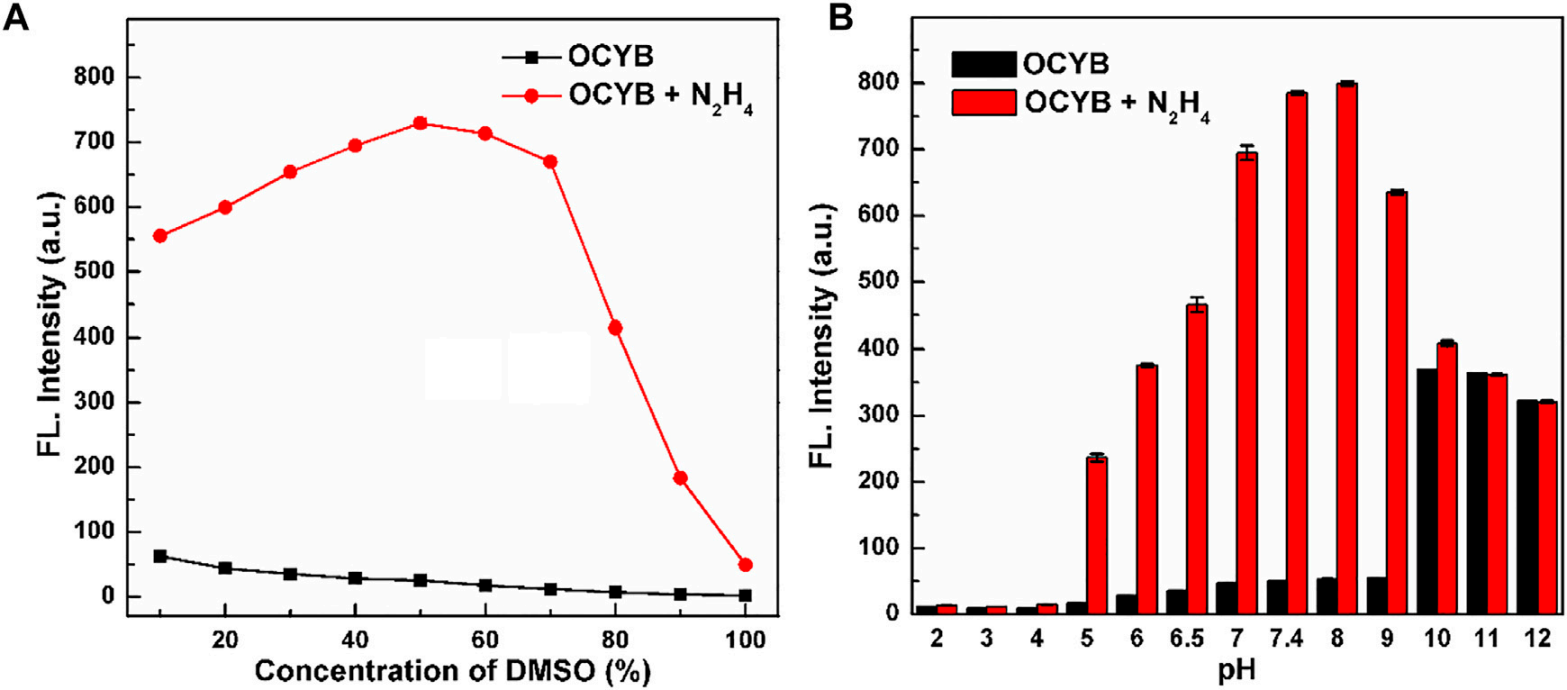
Ratiometric response (I_458 nm_/I_330 nm_) of OCYB (10 μM) without and with N_2_H_4_ (200 μM) in HEPES buffer solution containing **(A)** 10–100% DMSO and **(B)** at different pH with DMSO = 50%. Data are shown as the mean ± SE (bars) (*n* = 3).

The reaction conditions between the probe OCYB and N_2_H_4_ were optimized to obtain the best experimental conditions. The highest fluorescence intensity of the OCYB-N_2_H_4_ system was established by testing different DMSO concentrations, resulting in the discovery of the best-matched solvent ratio for the reaction between OCYB and N_2_H_4_ (Figure 1A). The influence of pH on the detection system was also studied. As shown in Figure 1B, the probe showed essentially no change in fluorescence intensity (I_458nm_) at pH 2-9, while the OCYB-N_2_H_4_ system showed a significant emission signal in the pH 7-9 range following reaction with N_2_H_4_ (200 μM). Considering the application of OCYB *in vivo* and *in vitro*, we chose pH 7.4 according to its physiological conditions. Based on the above reaction conditions, we further studied the effect of the reaction time between OCYB and N_2_H_4_. The fluorescence ratio response of the reaction system rose as the reaction time grew after adding N_2_H_4_ to the OCYB probe solution. Because the system remained stable after 1.5 h, the reaction time was fixed to 1.5 h in the following trials (Supplementary Figure S4).

### Selectivity and Anti-interference of OCYB Against N_2_H_4_

**FIGURE 2:**
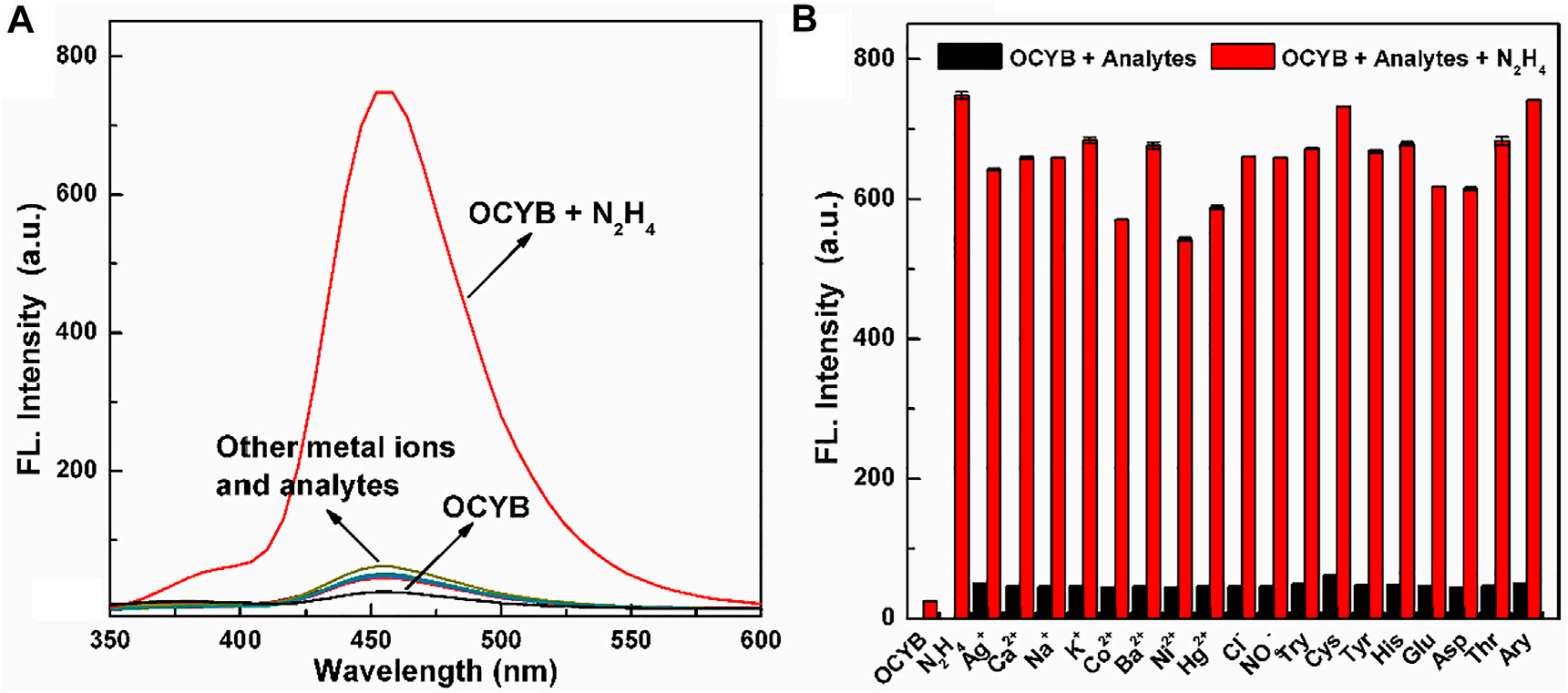
Fluorescence spectra **(A)** of OCYB (10 μM) in the presence of different interference analytes. **(B)** The fluorescence intensity of OCYB and N_2_H_4_ (200 μM) in the presence of other interference analytes (200 μM) in DMSO-HEPES buffer solution (1:1, v/v, pH 7.4, λ_ex_ = 330 nm). Data are shown as the means ± SE (bars) (*n* = 3).

We did additional research after determining that the OCYB probe can react with N_2_H_4_ using the preceding experiments and identifying the reaction’s ideal parameters. To determine whether the OCYB probe can specifically recognize N_2_H_4_, we selected a series of ions (Ag^+^, Ca^2+^, Na^+^, K^+^, Co^2+^, Ba^2+^, Ni^2+^, Hg^2+^, NO_3_^−^, Cl^−^) and compounds (Try, Cys, Tyr, His, Glu, Asp, Thr, Ary) to study the effect on the DDPB probe. With the addition of ions and various analytes, the fluorescence characteristics of the OCYB-N_2_H_4_ probe did not change. But when N_2_H_4_ was added, the fluorescence spectrum of the OCYB-N_2_H_4_ system was significantly enhanced (Figure 2A). Only the fluorescence intensity of the OCYB-N_2_H_4_ combination is dominating at an excitation wavelength of 330 nm, whereas other investigations only reveal the smallest fluorescence response in the presence of OCYB. The preceding data show that the OCYB probe detects N_2_H_4_ with good selectivity.

Subsequently, the anti-interference ability of the OCYB probe was continued. The fluorescence intensity of the OCYB probe changed significantly with the addition of N_2_H_4_ and the coexistence of anions, cations and typical bioanalytics did not effect on the detection of N_2_H_4_ by probe OCYB (Figure 2B). Thus, the OCYB probe has good specificity and strong resistance to interference for the detection of N_2_H_4_ and can be used in complex systems or environments.

### Response of OCYB to N_2_H_4_

**FIGURE 3:**
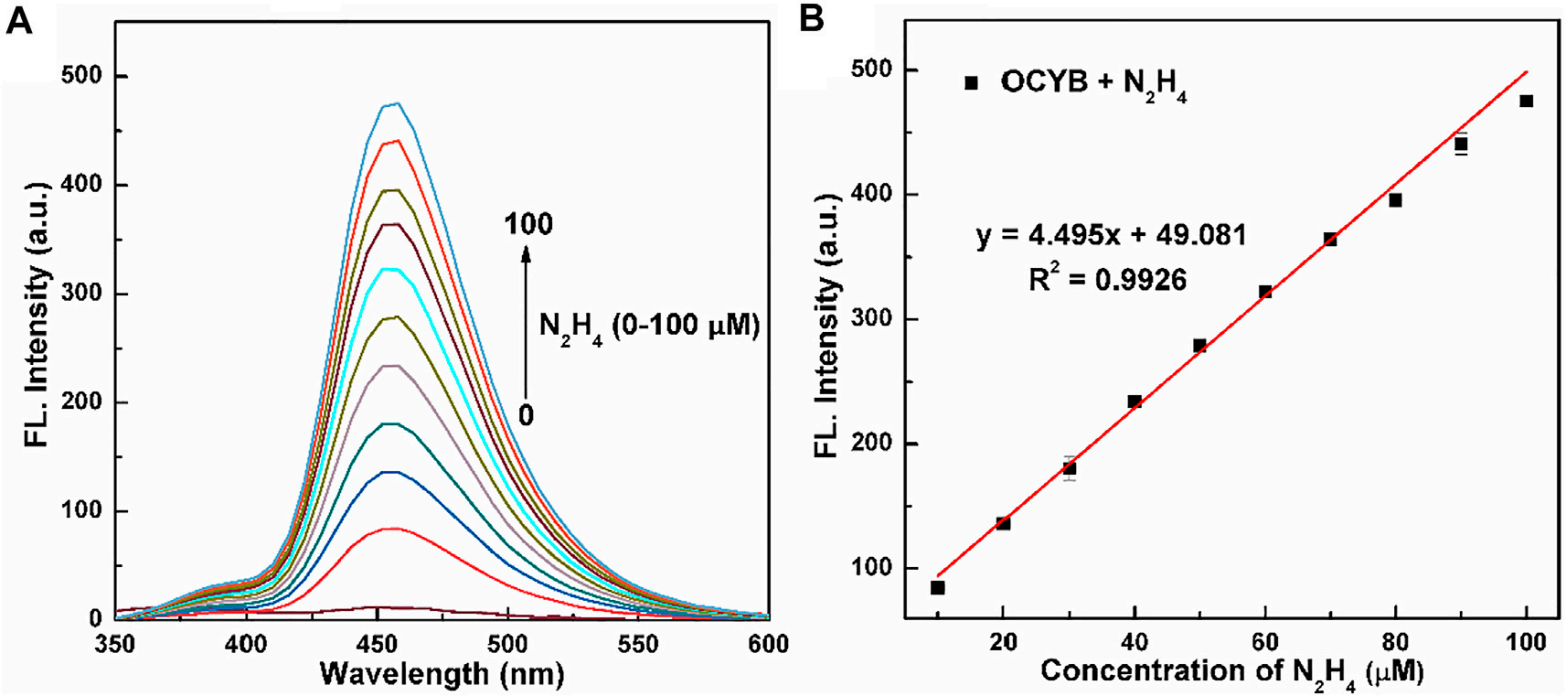
Fluorescence spectra **(A)** of OCYB (10 μM) with a series of concentrations of N_2_H_4_ (0–100 μM) in DMSO-HEPES buffer solution (1:1, v/v, pH 7.4, λ_ex_ = 330 nm). The linear relationship between the fluorescence intensity **(B)** of OCYB and the N_2_H_4_ concentration (0–100 μM). Data are shown as the mean ± SE (bars) (*n* = 3).

The sensitivity and linear range of the probe OCYB were further investigated using N_2_H_4_ fluorescence titration experiments, as shown in Figure 3a, which recorded the variation of the probe OCYB (10 μM) fluorescence emission spectrum in DMSO-HEPES buffer (1:1, v/v, pH 7.4) in the range of 0–100 μM of N_2_H_4_ concentration. The probe OCYB did not fluoresce on its own, but the fluorescence intensity at 458 nm grew dramatically as the N_2_H_4_ concentration was increased. In the concentration range of 0–100 μM, there is a good linear relationship between the fluorescence intensity y of the probe OCYB and the concentration x of N_2_H_4_, as shown in Figure 3B, with the regression equation y = 4.4951x + 49.0814 (*R* ^2^ = 0.9926), indicating that the probe OCYB can quantitatively detect N_2_H_4_ over a wide range. According to the equation LOD = 3N/S (LOD, N, and S are the detection limit, noise (mV), and slope, respectively), the LOD of the OCYB probe was calculated to be 78 nM, which is much lower than the US EPA’s threshold limit (0.31 μM), indicating that the probe OCYB has a good detection sensitivity for N_2_H_4_.

### Reaction Mechanism

**FIGURE 4:**
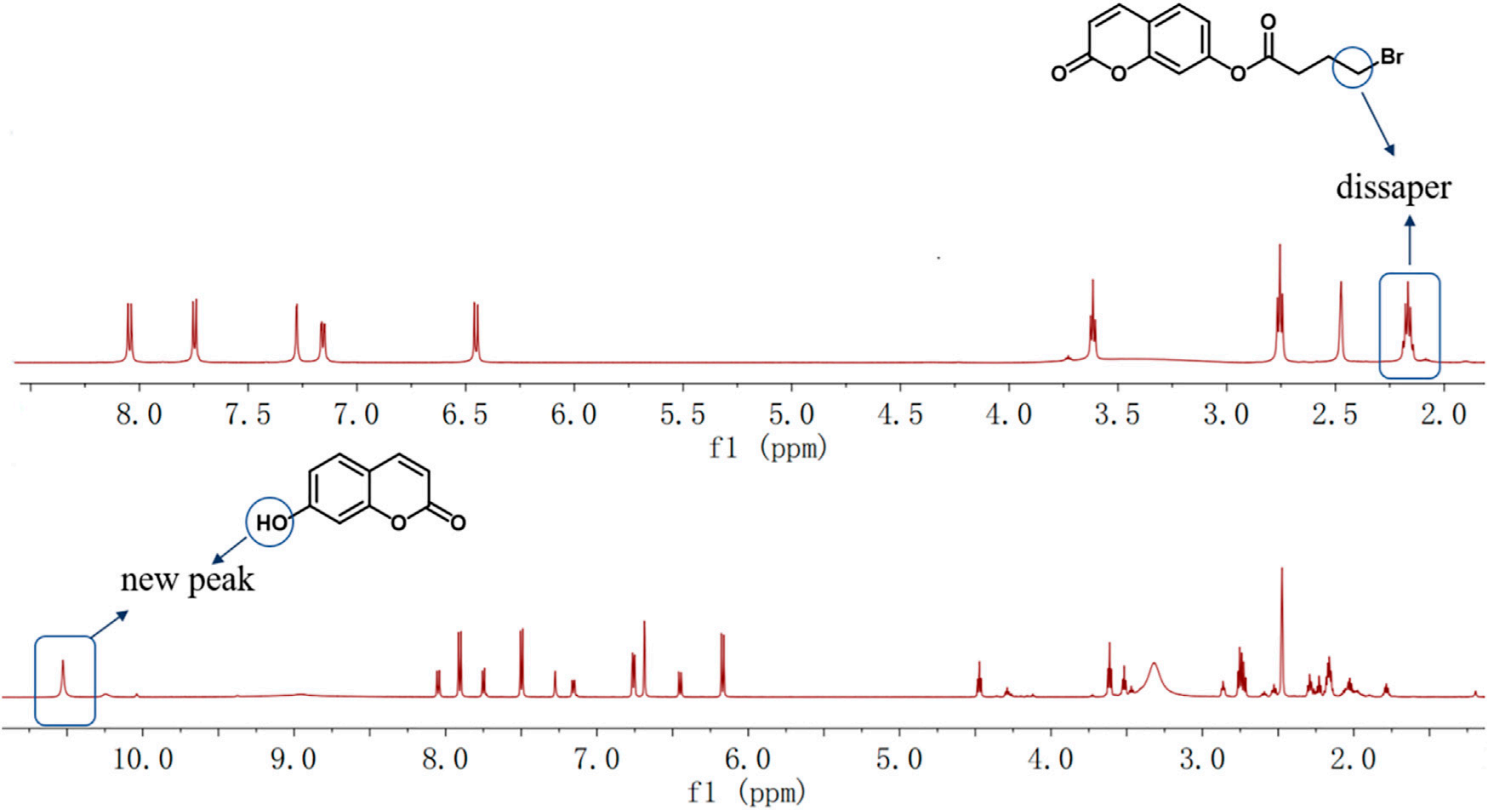
^1^H NMR spectra of OCYB before and after adding N_2_H_4_.

Bromoester derivatives are known to react with N_2_H_4_ by nucleophilic substitution of bromine groups and nucleophilic addition of ester carbon groups, followed by intramolecular cyclization to release fluorophores. For understanding the reaction mechanism of the probe OCYB on N_2_H_4_, we compared the ^1^H NMR spectra of OCYB and OCYB-N_2_H_4_ system (Figure 4). The hydrogen peak near the bromine atom on the 4-bromobutyryl group arises at 2.0–2.4 ppm, as illustrated in the image. The hydrogen peak near the bromine atom vanishes once the reaction of OCYB with N_2_H_4_ is completed, and a new peak at 10.5 ppm appears. These results suggest that OCYB undergoes a substitution-cyclization reaction triggered by N_2_H_4_ that releases fluorescent groups, which enhances the fluorescence signal. To corroborate the aforesaid mechanism, we used HRMS to investigate the OCYB-N_2_H_4_ product (Supplementary Figure S5) and found a signal at m/z=163.0386, which is nearly identical to the theoretical molecular weight of 7-hydroxycoumarin ([M+H]^+^ = 163.0317). The above findings suggest that OCYB experienced a substitution process triggered by N_2_H_4_, resulting in the release of the 7-hydroxycoumarin fluorophore and a shift in the fluorescence signal (Scheme 1B).

### Computational Study of OCYB

**FIGURE 5:**
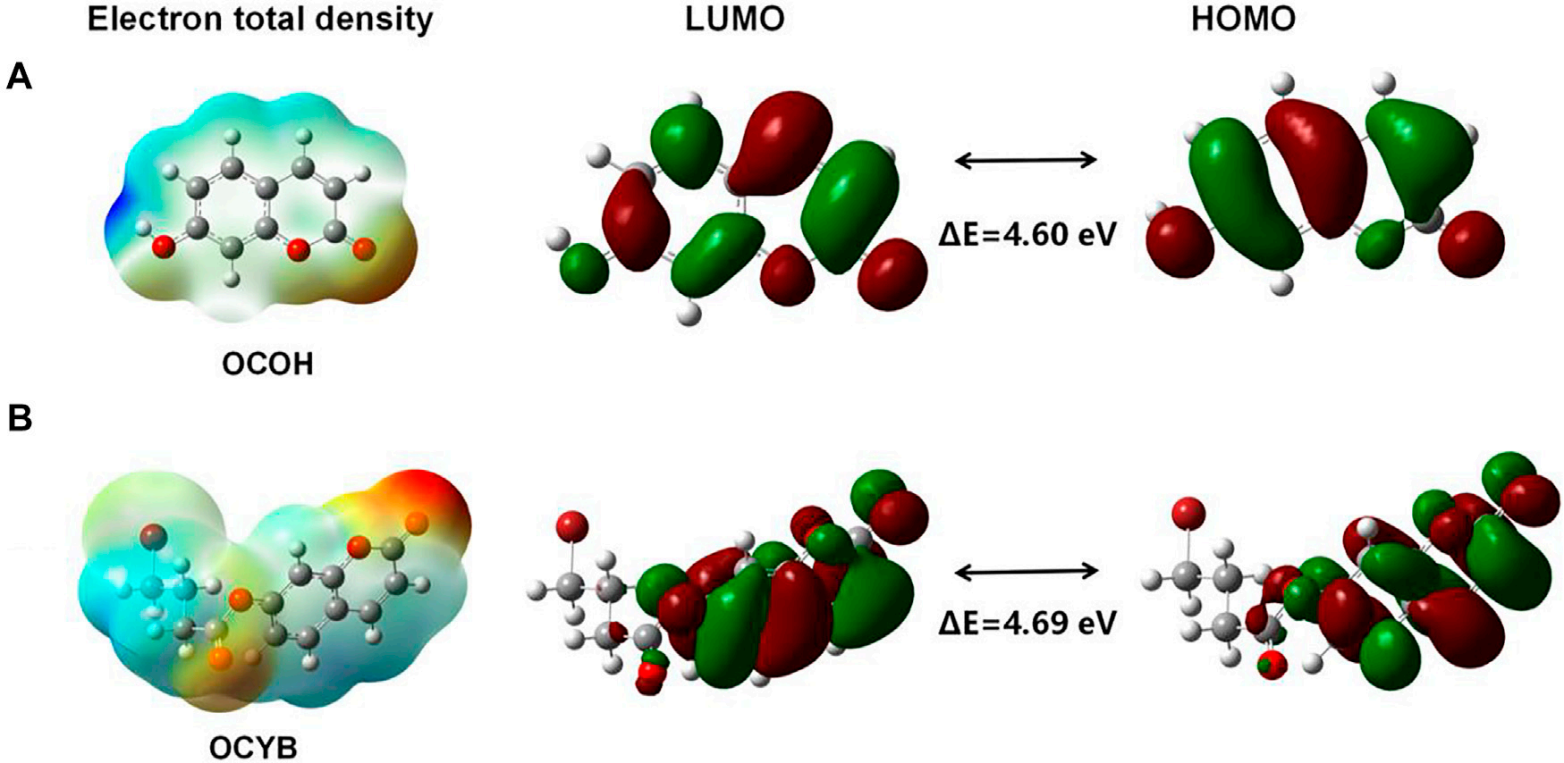
Quantum chemical calculations of OCOH **(A)** and OCYB **(B)**.

The molecular structure and charge distribution of OCYB and its product OCOH were simulated using density general function theory (DFT) calculations (B3LYP/6–31*G) to gain insight into the sensing mechanism of OCYB on N_2_H_4_. The optimized molecular structures of OCOH and OCYB are shown in Supplementary Figures S6, S7. The fluorescence enhancement of OCYB induced by N_2_H_4_ is mainly summarized in two aspects. The phenolic hydroxyl group on OCYB belongs to the electron-absorbing group (acceptor) and the 4-bromobutyryl group as the electron-donating group (donor) forms a π-π conjugation system. The intramolecular transfer of electrons from the donor to the acceptor enhances the fluorescence, while the modification of the hydroxyl group on OCOH by the 4-bromobutyryl group blocks this process, resulting in a fluorescence quenching (Figure 5). The donor in the fluorophore reacts with the target molecule with an enhanced electron absorption capacity of the acceptor and a change in the LUMO/HOMO energy level difference of the fluorophore (4.69–4.60 eV). The reaction of OCYB and N_2_H_4_ was also shown to be based on an intramolecular charge transfer (ICT) mechanism, which results in increased fluorescence of the probe OCYB.

### Practical Applications

Water samples were gathered from rivers near parks and schools in Hefei, Anhui, China, as test water samples for the assessment and analysis of N_2_H_4_ using OCYB to evaluate the probe’s practical application in detecting N_2_H_4_. The OCYB probe was introduced to a water sample having a 10 μM N_2_H_4_ gradient concentration. The fluorescence spectra of OCYB-N_2_H_4_ in water samples were recorded, and a standard addition curve was created using the specific fluorescence intensity as the y-axis and the amount of N_2_H_4_ supplied as the x-axis (Supplementary Figure S8). According to Table 1, N_2_H_4_ detection fluorescence recoveries ranged from 80 to 104%, showing that the probe OCYB was successful in detecting N_2_H_4_ in actual solutions.

**TABLE 1:** Determination of N_2_H_4_ in real water samples.

Samples	Concentration of N_2_H_4_		Recovery (%)
	Added Amount (mg/L)	Recovered Amount (mg/L)	
Pure Water (Hefei, Anhui)	0.10	0.09 ± 0.01	90
	0.50	0.52 ± 0.01	104
	1.00	1.02 ± 0.02	102
Blackpool Dam Water (Hefei, Anhui)	0.10	0.09 ± 0.01	90
	0.50	0.47 ± 0.03	94
	1.00	0.99 ± 0.01	99
Moat water (Hefei, Anhui)	0.10	0.10 ± 0.02	100
	0.50	0.46 ± 0.01	92
	1.00	0.93 ± 0.04	93
Pond Water (Anhui Agricultural University)	0.10	0.08 ± 0.02	80
	0.50	0.48 ± 0.01	96
	1.00	0.97 ± 0.02	97

a The above water sample is taken at multiple points, and the sampling point is not less than 5. Data are mean ± SE (bars) (*n* = 3).

### Cellular Experiments

**FIGURE 6:**
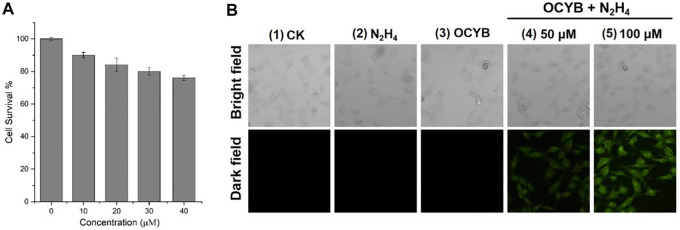
**(A)** Cell survival of different concentration of OCYB; **(B)** Fluorescence images for HeLa cells (1: blank control. 2: N_2_H_4_. 3: OCYB. 4 and 5: HeLa cells were treated with OCYB and then incubated with N_2_H_4_, Ex = 375 nm).

The survival rate of HeLa cells treated with MTT colorimetric assay at varying doses of OCYB solution for 24 h was used to determine the survivability of the probe OCYB for *in vivo* research. The survival rate of HeLa cells in all four groups decreased with increasing probe concentration (Figure 6A). The greatest concentration of OCYB used was 40 μM, and HeLa cells survived 76% of the time at this concentration, with the average survival rate of the four groups being 83%. The results of the foregoing experiments show that the probe OCYB has low cytotoxicity and can be employed *in vivo* studies.

N_2_H_4_ in the environment, ingested by creatures through the mouth, nose, or skin surface, has powerful toxic effects on species, and if ingested by humans, can cause serious harm to the human system. Therefore, the study of fluorescence imaging of N_2_H_4_ in living cells is an important guideline for both biological research and the development of the pharmaceutical industry. HeLa cells were used to test the fluorescence imaging ability of OCYB in living cells in this study (Figure 6B). No fluorescence was recorded in HeLa cells from blank control, only OCYB, and containing N_2_H_4_ under the fluorescence channel. Correspondingly, after treatment with OCYB and then incubation with N_2_H_4_ (50 μM, 100 μM), the distribution of strong green fluorescence in HeLa cells could be seen under fluorescence. It can also be seen that the fluorescence intensity of the group 5 at N_2_H_4_ (100 μM) is higher, and the image is clearer. This is due to the release of more fluorescent groups due to the reaction of OCYB with the added exogenous N_2_H_4_. The intracellular fluorescence changes caused by N_2_H_4_ imply that OCYB penetrates HeLa cells well and is responsive to extracellular and extracellular sources of N_2_H_4_, allowing N_2_H_4_ imaging in living HeLa cells.

### Zebrafish Imaging

**FIGURE 7:**
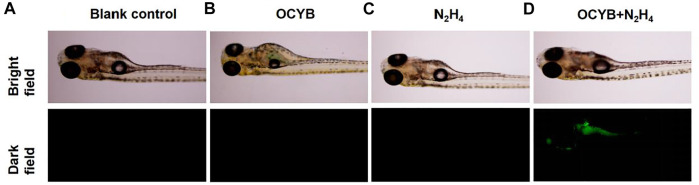
Fluorescence images of zebrafish: **(A)** blank control. **(B)** OCYB. **(C)** zebrafish incubated with N_2_H_4_ (100 μM) for 30 min **(D)** zebrafifish samples pretreated with N_2_H_4_ (100 μM) for 30 min before incubation with OCYB (20 μM) for 30 min (Ex = 375 nm).

Because zebrafish and human genes have an 87% genetic similarity, zebrafish were chosen as the subject for *in vivo* fluorescence imaging of hydrazine. As shown in Figure 7, there was no significant differential change in zebrafish between the four treatment conditions under bright field conditions. However, zebrafish incubated with N_2_H_4_ (100 μM) under darkfield conditions and then treated with OCYB for 30 min showed a strong bright green fluorescence effect. The green fluorescence is mainly found in the head, stomach, respiratory tract and digestive tract of zebrafish. The experimental results showed that the fluorescent probe OCYB could enter zebrafish and visualize the distribution of N_2_H_4_ in the organism. It is also further demonstrated that OCYB presents low toxicity with little biological hazard.

## Conclusion

In conclusion, a coumarin-based hydrazine-specific detection fluorescent probe OCYB based on the ICT mechanism was synthesized in this paper. The key features of OCYB include easy availability of raw materials, low cost, large Stokes shift (128 nm), low detection limit (78 nM), low toxicity, good cell permeability, and high selectivity. HRMS, NMR, and DFT calculations were used to verify the mechanism of the reaction between OCYB and N_2_H_4_. According to the results of the practical application, OCYB can be used to detect N_2_H_4_ in real water samples. Furthermore, due to its low cytotoxicity and good biocompatibility, OCYB has also been successfully applied for imaging endogenous N_2_H_4_ in live HeLa cells and zebrafish. The present study provides important information for the visual detection of environmental contaminants.

## Data Availability

The original contributions presented in the study are included in the article/Supplementary Material, further inquiries can be directed to the corresponding authors.
