# Supplementary material for: An ICT-Based Coumarin Fluorescent Probe for the Detection of Hydrazine and Its Application in Environmental Water Samples and Organisms
Source: Front Bioeng Biotechnol. 2022 Jun 14;10:937489. doi: 10.3389/fbioe.2022.937489 (PMC9237640; doi:10.3389/fbioe.2022.937489)
Supplement: Supplementary file 1 [file DataSheet1.docx]

Supplementary Data

**An ICT-based coumarin fluorescent probe for the detection of hydrazine and its application in environmental water samples and organisms**

Xina Liu^1†^, Meiqing Zhu^2†^, Fugang Fan^1^, Chenyang Xu^1^, Panpan Chen^1^, Yi Wang^1, *^, Dongyang Li^3, *^

^1^ Anhui Provincial Key Laboratory of Quality and Safety of Agricultural Products, College of Resources and Environment, Anhui Agricultural University, Hefei 230036, China

^2^ School of Chemical and Environmental Engineering, Anhui Polytechnic University, Wuhu, Anhui 241000, China

^3^ Laboratory of Agricultural Information Intelligent Sensing, College of Biosystems Engineering and Food Science, Zhejiang University, Hangzhou, Zhejiang, 310058 China

*Corresponding Author

Email address:

wangyi1987@cau.edu.cn (Prof. Yi Wang)

[dylee@zju.edu.cn](mailto:dylee@zju.edu.cn) (Prof. Dongyang Li)

^†^Contributed equally to the work

**List of Figures**

**Figure S1.** ^13^C-NMR spectra of OCYB.

**Figure S2.** ^1^H-NMR spectra of OCYB.

**Figure S3.** LC-HRMS spectra of OCYB.

**Figure S4.** Time-dependent fluorescence intensities of OCYB (10 μM) with N_2_H_4_ (200 μM).

**Figure S5.** LC-HRMS spectrum of the products of the reaction between OCYB and N_2_H_4_.

**Figure S6.** Optimized structure of probe OCYB.

**Figure S7.** Optimized structure of probe OCOH.

**Figure S8**. Standard curves (b) of different concentrations (0.1, 0.5, 1.0, 1.5, 2.0 mg/L) of N_2_H_4_ in OCYB solution.

**Figure S9**. Cytotoxicity of OCYB (0, 10, 20, 30, 40 μM) evaluated on living HeLa cells by the standard CCK-8 assay.


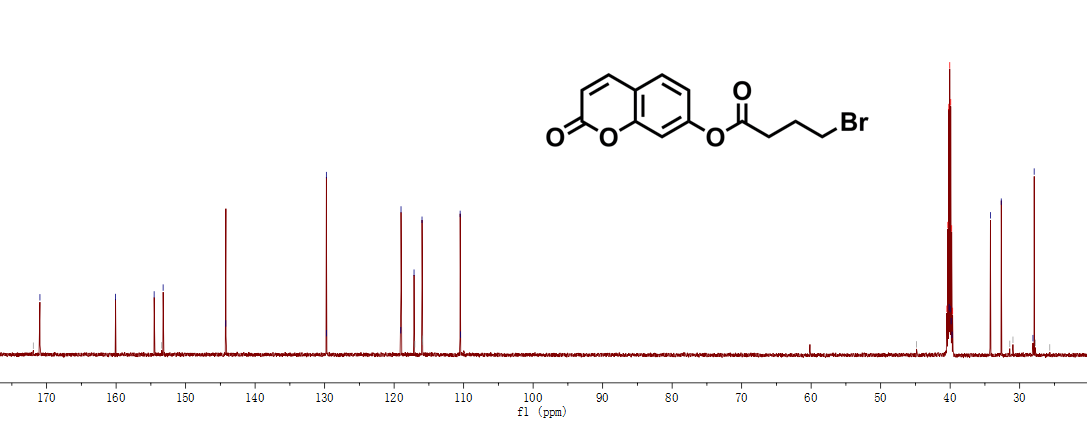


**Figure S1.** ^13^C-NMR spectra of OCYB


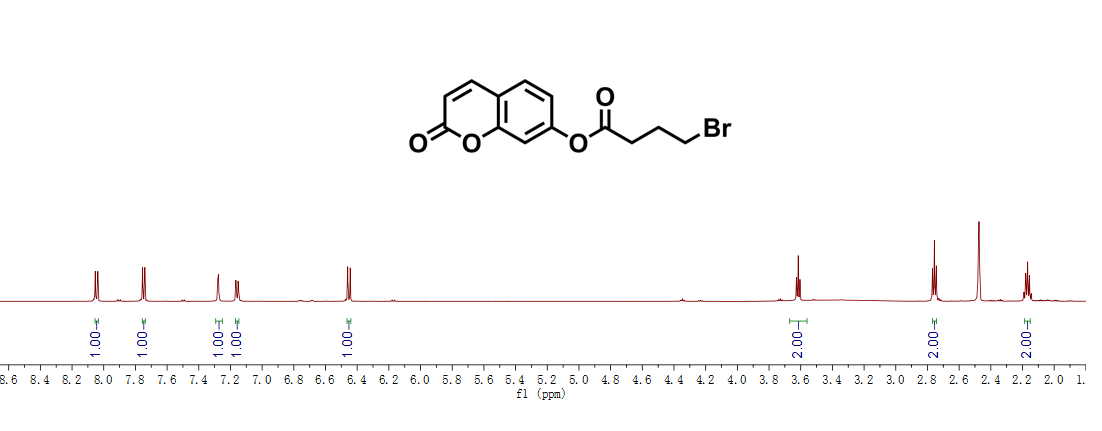


**Figure S2.** ^1^H-NMR spectra of OCYB.

**
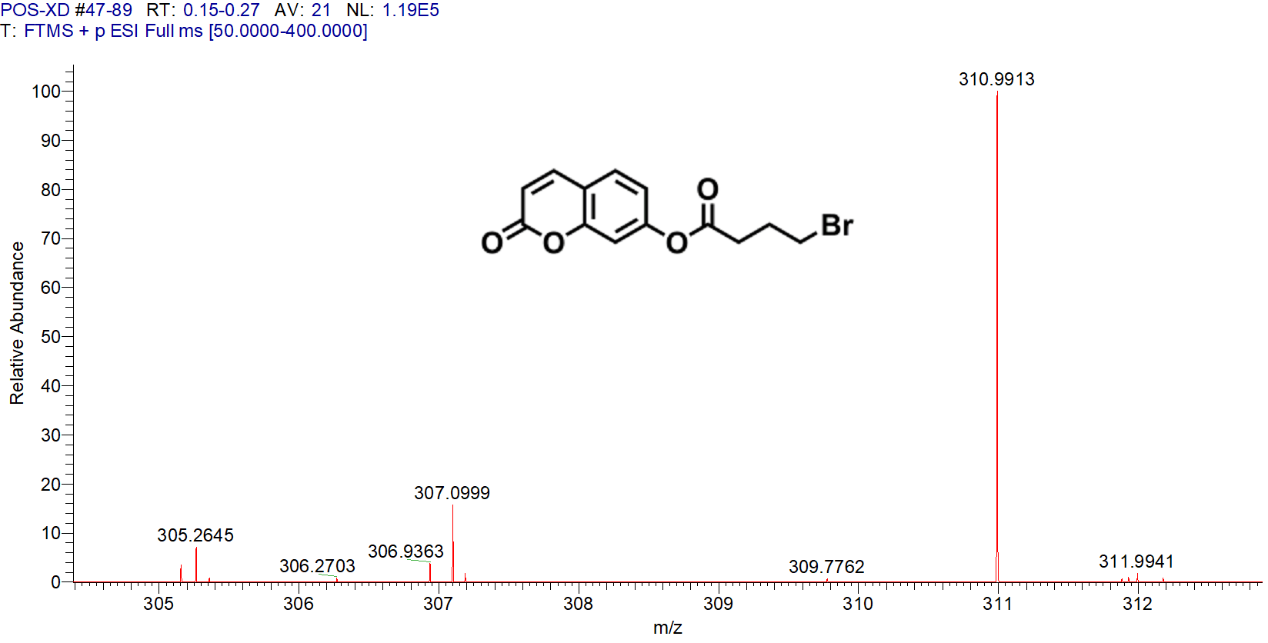
**

**Figure S3.** LC-HRMS spectra of OCYB.





**Figure S4.** Time-dependent fluorescence intensities of OCYB (10 μM) with N_2_H_4_ (200 μM).


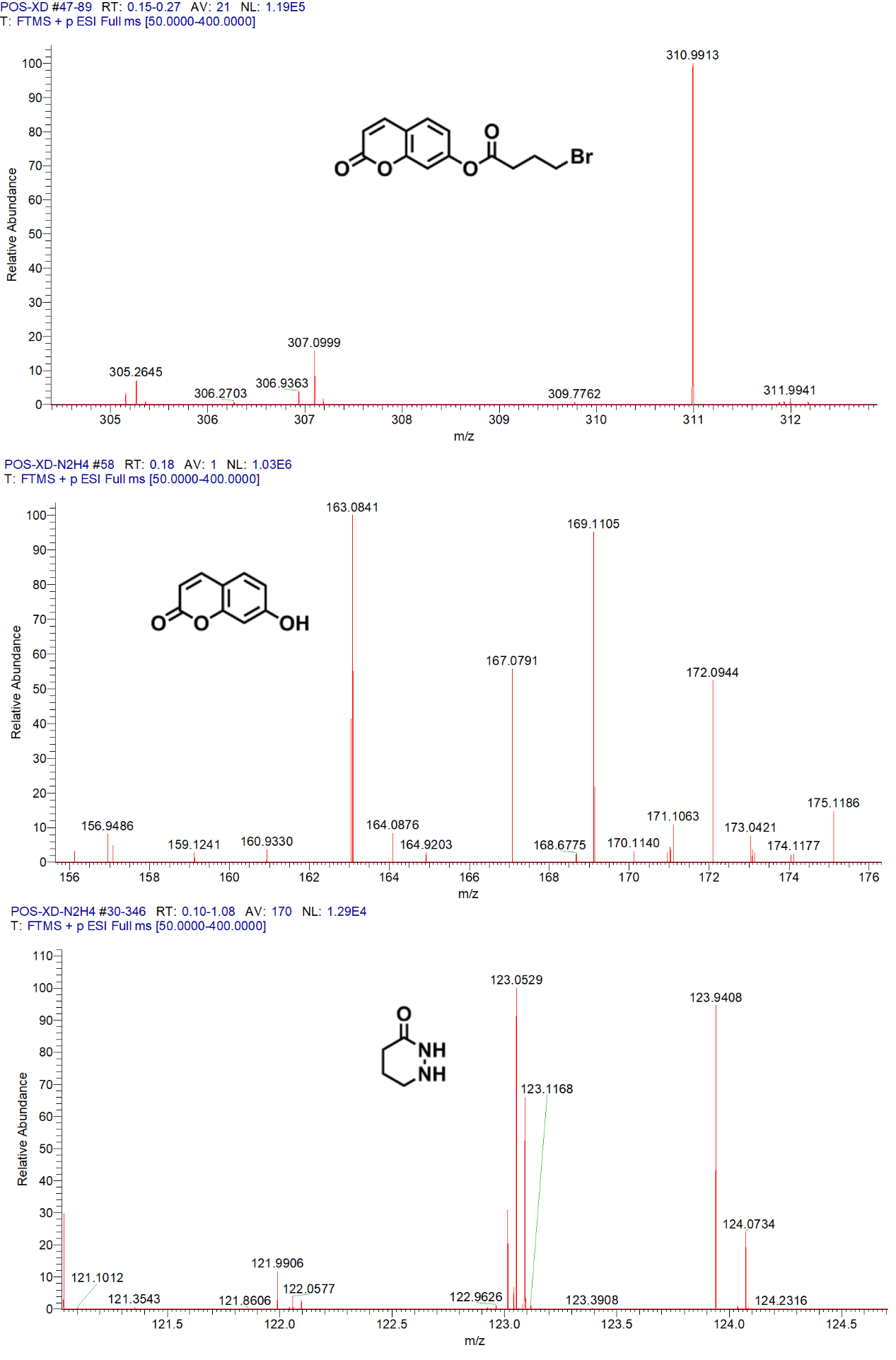


**Figure S5.** LC- HRMS spectrum of the products of the reaction between OCYB and N_2_H_4_.


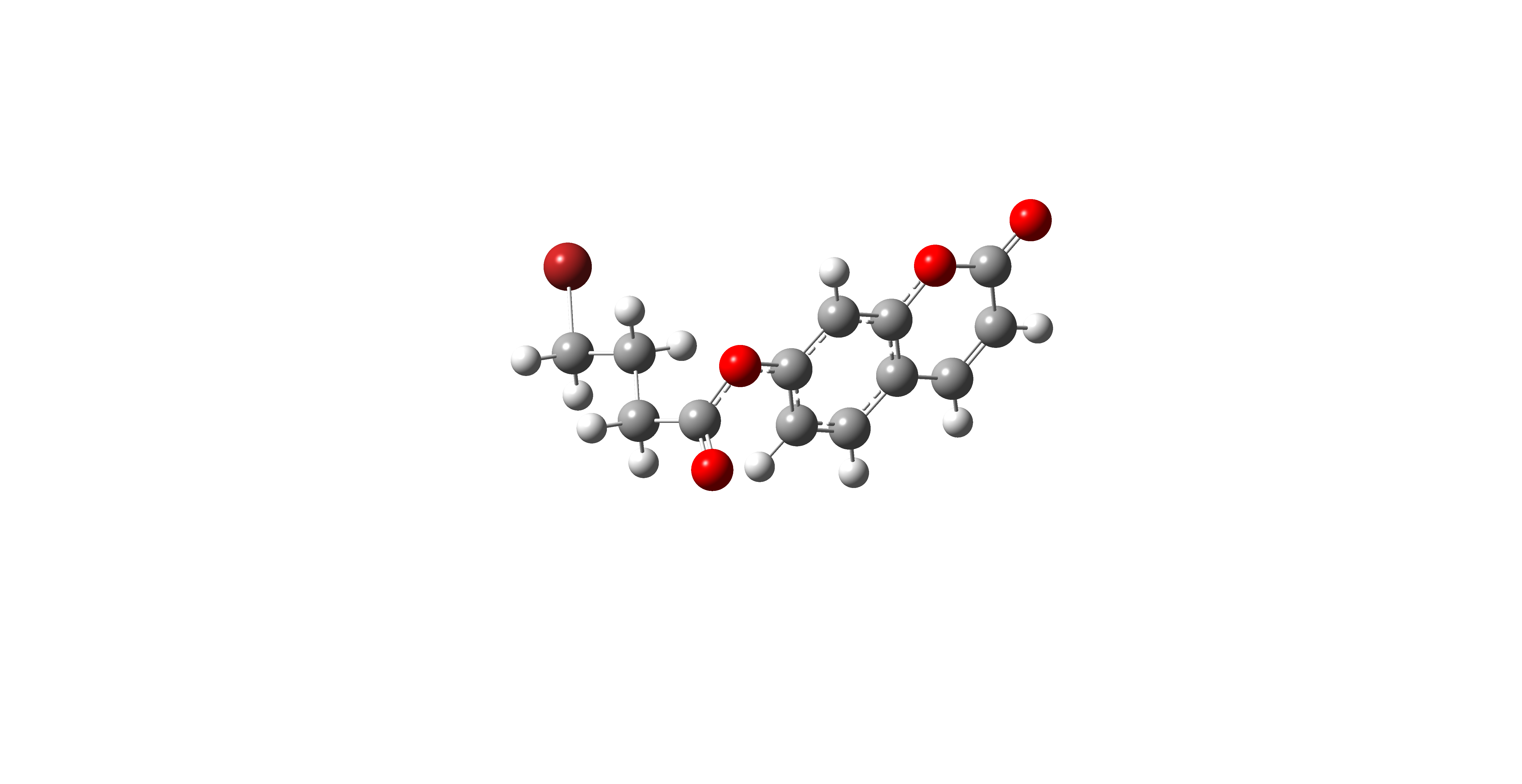


**Figure S6.** Optimized structure of probe OCYB

C -0.3354 0.76254 -0.93112

C -0.33168 -0.63119 -0.93118

C 0.87961 -1.32303 -0.9312

C 2.08821 -0.62363 -0.9312

C 2.09006 0.77406 -0.9312

C 0.87228 1.46451 -0.93112

O 3.22097 -1.38814 -0.93125

C 4.48943 -0.7207 -0.93128

C 4.47773 0.77773 -0.93127

C 3.35242 1.4967 -0.93111

O 5.52597 -1.34106 -0.93131

O -1.50266 -1.31298 -0.93122

C -2.56003 -0.49309 -0.93119

C -3.15628 -0.03081 0.37566

C -2.37464 -0.63696 1.53374

C -2.97643 -0.17039 2.85271

Br -1.97615 -0.94609 4.33472

O -3.03734 -0.12293 -1.97733

H -1.28956 1.30988 -0.93107

H 0.88272 -2.42303 -0.93122

H 0.86637 2.5645 -0.93106

H 5.44041 1.30995 -0.93139

H 3.37916 2.59638 -0.93092

H -3.10424 1.07942 0.43445

H -4.2188 -0.35694 0.43444

H -2.42668 -1.74719 1.47496

H -1.31211 -0.31083 1.47497

H -2.92439 0.93984 2.9115

H -4.03895 -0.49652 2.91149


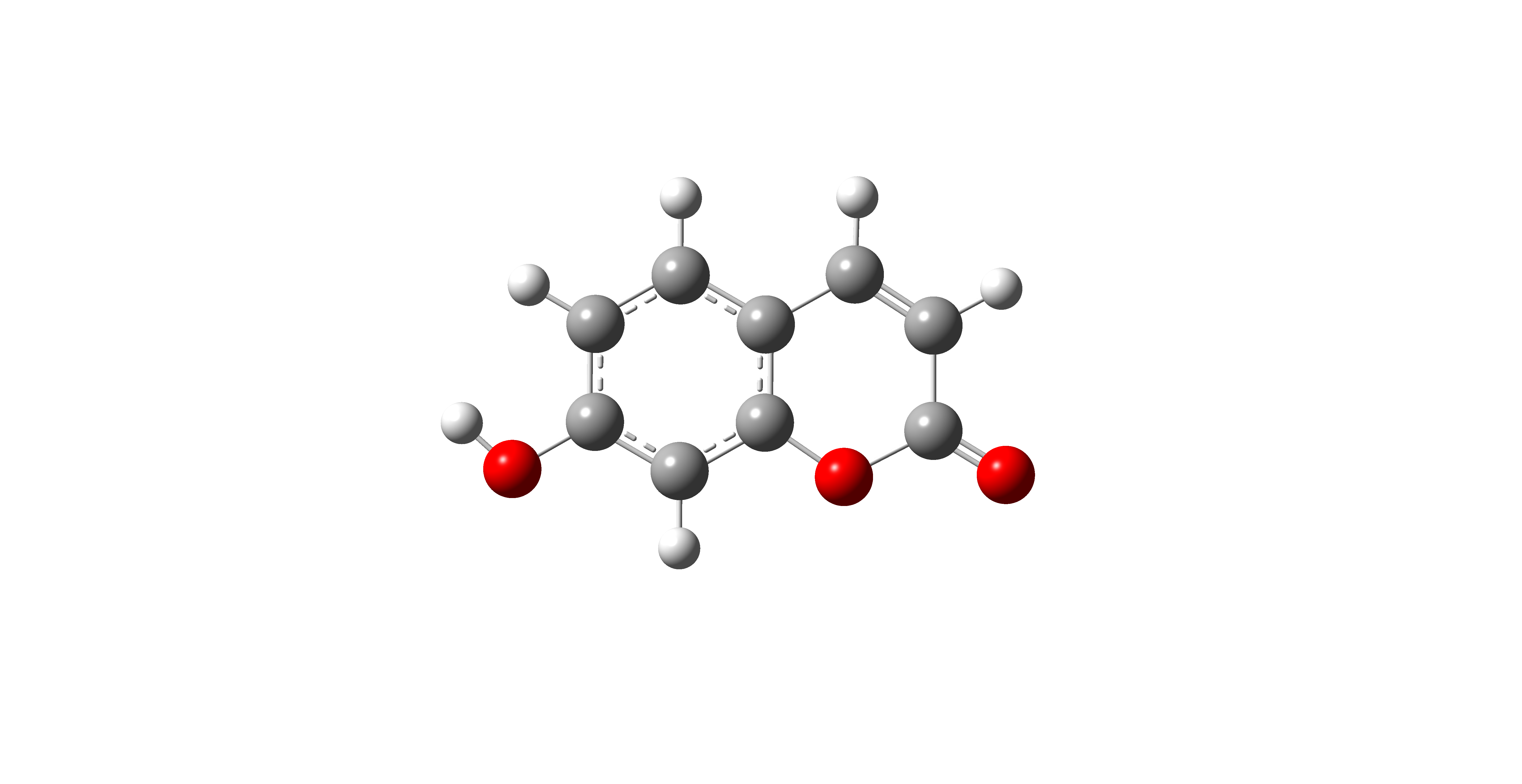


**Figure S7.** Optimized structure of probe OCOH

C -2.10644 0.61658 0.00007

C -2.10272 -0.77715 0.

C -0.89143 -1.46899 -0.00002

C 0.31718 -0.76959 -0.00002

C 0.31902 0.6281 -0.00002

C -0.89876 1.31856 0.00006

O 1.44993 -1.53409 -0.00007

C 2.71839 -0.86666 -0.00009

C 2.7067 0.63177 -0.00008

C 1.58139 1.35074 0.00008

O 3.75493 -1.48702 -0.00012

O -3.2737 -1.45894 -0.00003

H -3.0606 1.16392 0.00011

H -0.88832 -2.56899 -0.00004

H -0.90466 2.41854 0.00013

H 3.66937 1.16399 -0.00021

H 1.60812 2.45042 0.00027

H -3.99841 -0.81119 -0.00001





**Figure S8.** Standard curves (b) of different concentrations (0.1, 0.5, 1.0, 1.5, 2.0 mg/L) of N_2_H_4_ in OCYB solution.
